# Wellbeing for staff in UKCRC-registered Clinical Trials Units: Development of the Flourishing As Clinical Trial Staff (FACTS) guidance: a mixed-methods study

**DOI:** 10.1016/j.conctc.2025.101556

**Published:** 2025-10-03

**Authors:** Sophie S. Hall, Evgenia Riga, Eleanor J. Mitchell, Louise Thomson, Jodi Taylor, Lucy Carr, Pamela Hagan, Kirsty Sprange

**Affiliations:** a Nottingham Clinical Trials Unit, School of Medicine, University of Nottingham, Nottingham, UK; b Faculty of Medicine and Health Sciences, University of Nottingham, Nottingham, UK; c Sheffield Clinical Trials Research Unit, University of Sheffield, Sheffield, UK; d Bristol Trials Centre, University of Bristol, Bristol, UK

**Keywords:** Clinical trials, Clinical trial staff, Flourishing, Workplace wellbeing, Employee wellbeing, Eudaimonic wellbeing, Job satisfaction

## Abstract

**Background:**

Evaluating healthcare interventions in clinical trials requires a skilled workforce. However, the demands of developing and running clinical trials make recruiting and retaining staff challenging. Flourishing, which focuses on positive aspects of well-being, may help staff manage these demands. This study introduces the Flourishing As Clinical Trial Staff (FACTS) guidance, offering practical strategies to support staff working in UK Clinical Research Collaboration (UKCRC) Clinical Trials Units (CTUs), to thrive at work.

**Methods:**

Building upon findings from a national survey of staff working in UKCRC CTUs, a three-phase consensus-based approach was used to develop recommendations to support flourishing in clinical trial staff; (1) focus groups with staff (n = 24), (2) a consensus survey (n = 21) and (3) a consensus workshop (n = 15).

**Results:**

The focus groups identified strategies for supporting CTU staff to flourish, including factors relating to the *environment* (e.g., flexible working)*; interpersonal communication* (e.g., supportive colleagues), *growth* (e.g., protected training time) and *acknowledgement* of everyone's contributions. These strategies were developed into 67 wellbeing recommendations which were further evaluated in a consensus survey and workshop. Following this, 61 recommendations were endorsed for inclusion in the guidance.

**Conclusions:**

The FACTS guidance includes recommendations to support UKCRC CTU staff to flourish in their work and are likely to apply more broadly to research institutions conducting clinical trials. The recommendations provide a foundation for CTUs to review and adapt to their local needs over time. Implementing these recommendations may prove beneficial for increasing job satisfaction and commitment, which is likely to facilitate efficient trial delivery.

## 1 Background

Workplace wellbeing is recognised by the World Health Organisation as a multi-dimensional concept, incorporating the interconnectedness of physical, mental, and social factors that contribute to overall wellbeing [1]. Workplace wellbeing influences workplace productivity [2,3] and is therefore important to supporting a sustainable and productive society. Clinical trial staff are integral to the development of innovative healthcare solutions to address global health challenges. The design and conduct of clinical trials rely upon the expertise, dedication, and coordinated efforts of skilled staff members throughout the entire research process. It is vital that we support clinical trial staff to flourish in their role. Here we report the development of workplace wellbeing guidance to promote wellbeing in clinical trial staff, with the aim of developing a satisfied, fully functioning workforce who can contribute to scientific advancements and healthcare developments. The guidance was co-developed with staff from UK Clinical Research Collaboration (UKCRC)-registered Clinical Trials Units (CTUs), who are the primary target audience. However, the principles outlined are likely relevant to those working in clinical trials across other settings as well.

Recruitment and retention of clinical trial staff is a global issue. Although there is limited published research in this area, directors of clinical research units, including the UKCRC and cooperative groups like the Southwest Oncology Group (SWOG) Cancer Research Network, have informally discussed the challenges of recruiting and retaining qualified trial staff [[4], [5], [6], [7], [8], [9], [10], [11], [12]]. Recruitment and retention challenges have a negative impact on virtually all aspects of clinical trial design and delivery and are likely to contribute to inefficiencies often associated with clinical research [[13], [14], [15], [16]]. Indeed, common reasons that are reported for lack of efficiency include issues relating to poor management of an unmotivated and inexperienced trial team, and insufficient trial staff in place [[16], [17], [18], [19]]. Inefficient clinical research leads to financial as well as ethical implications [20].

In the UK many clinical trials are conducted through a CTU. A successful CTU requires a broad range of skilled experts (e.g., trial management, statistical expertise, data management and programming, trial design, quality assurance, administration) to work collaboratively with a common goal [21]. Trials are delivered in a dynamic, time-pressured environment, whereby staff must readily adapt to recent scientific advancements/priorities, unexpected events, and societal shifts (e.g., remote working) [22]. In addition, clinical trials often take several years to design, set-up and complete, requiring sustained motivation and resilience amongst staff which can be further tested when challenges occur. Many of these demands are unique to clinical trial research, in contrast to research studies typically led by smaller research teams. The latter often have fewer demands for the integration of a diversely skilled team and operate under less stringent regulatory requirements and standard operating procedures.

The demands associated with working in clinical trials underscore the critical need to support staff in cultivating resilience—enabling them to adapt and rebound effectively from challenges while maintaining optimal functioning over a sustained period. This approach is congruent with the psychological framework of eudaimonic wellbeing, also referred to as flourishing [23], but is in contrast to typical discussions around workplace wellbeing which often centre on concepts such as stress and anxiety [24,25], impacting turnover intentions and organisational commitment [26,27].

Flourishing is rooted in Ryff's construct of psychological wellbeing. It encompasses six dimensions—self-acceptance, positive relations with others, autonomy, environmental control, purpose in life, and personal growth [28]. It emphasises individual development through engagement with life challenges, resilience-building, and the ability to fully function in various aspects of life [[28], [29], [30]], including the workplace [[31], [32], [33]]. This perspective underscores the significance of cultivating a work environment that facilitates thriving at both interpersonal levels (shaped by social and other external factors influencing the work experience) and intrapersonal levels (involving internal, personal factors) [34]. While there is a limited body of research applying this wellbeing framework to clinical trial staff, factors associated with interpersonal wellbeing, such as inadequate engagement and communication within trial teams, have been linked with trial inefficiencies [17]. Conversely, a flexible and supportive working environment that offers avenues for professional progress has been linked to heightened staff commitment to working on clinical trials [35]. Furthermore, considerations related to intrapersonal wellbeing, such as opportunities for personal and professional growth and a sense of meaningful contribution, have shown to mitigate turnover among trial staff [7].

We conducted a national survey [36] to explore the concept of flourishing in relation to job satisfaction among staff in UKCRC-registered trials units. Survey respondents (n = 484) reported ‘average’ levels of job satisfaction and work engagement but showed slightly lower levels of flourishing compared to other professions, along with moderate levels of turnover intention. We found notable associations between flourishing, job satisfaction, and turnover intention, underscoring the importance of a work environment that supports flourishing. Such support could enhance individual wellbeing and contribute to broader efficiencies within trials units by reducing turnover-related challenges.

Although Government-commissioned generic workplace guidance exists, including the Stevenson-Farmer Thriving and Work Report, National Institute for Health and Care Excellence (NICE) Mental Wellbeing at Work guidelines, and the Health and Safety Executive (HSE) guidelines [[37], [38], [39]], our recent research has uncovered a number of distinct needs that are not addressed in existing guidance [36].

The aim of this paper is to report the three-stage approach to develop the key recommendations which form the Flourishing As Clinical Trial Staff (FACTS) guidance. The recommendations are specifically tailored to the wellbeing challenges experienced by staff working in UK CTUs and draws upon a flourishing framework of wellbeing.

## 2 Methods

The study is reported using Consolidated criteria for reporting qualitative studies (COREQ) guidelines (see Supplementary Material 1).

### 2.1 Ethical approval

The study received ethical approval from the University of Nottingham Faculty of Medicine and Health Sciences Research Ethics Committee (FMHS 101–1022). Participants provided informed consent prior to any research procedures.

### 2.2 Recruitment

Participants were selected from respondents to a survey of UKCRC-registered CTU staff. The recruitment strategy for the survey has been previously reported [36]. Survey respondents who had agreed to be approached for further research activities were purposively selected to participate in the focus groups, based on their job role and their place of employment, to help ensure diversity and representativeness in the sample. Email invitations were sent to 24 participants.

### 2.3 Stage 1: Focus groups

***Participants.*** Twenty-four participants from 18 UKCRC-registered CTUs took part across five focus groups (see Table 1). All invited participants took part. To promote open speech, the groups were selected so that individuals working in similar roles/at a similar level were together. No focus group included colleagues from the same CTU.

**Table 1:** Participant characteristics.

	Focus Groups (n = 24)	Consensus Panel Survey (n = 18^a^)	Consensus Panel Workshop (n = 9^b^)
**Gender**			
Male	12.5 % (3)	11.1 % (2)	22.2 % (2)
Female	87.5 % (21)	88.9 % (16)	77.8 % (7)
**Ethnicity**			
White	95.8 % (23)	94.4 % (17)	88.9 % (8)
Asian	4.2 % (1)	5.6 (1)	11.1 % (7)
**Age**			
18–25 years	4.2 % (1)	5.6 % (1)	0 % (0)
26–36 years	41.7 % (10)	33.3 % (6)	33.3 % (3)
37–46 years	37.5 % (9)	44.4 % (8)	55.6 % (5)
47–56 years	16.7 % (4)	16.7 % (3)	11.1 % (1)
**Role in CTU**			
Trial Management	58.3 % (14)	61.1 % (11)	44.4 % (4)
Data/IT	12.5 % (3)	11.1 % (2)	11.1 % (1)
Statistician	4.2 % (1)	5.6 % (1)	0 % (0)
Quality Assurance	8.3 % (2)	11.1 % (2)	11.1 % (1)
CTU admin/operations	4.2 % (1)	5.6 % (1)	22.2 % (2)
Deputy/Senior Management	12.5 % (3)	5.6 % (1)	11.1 % (1)
**Employment Length in CTU**			
3–11 months	8.3 % (2)	11.1 % (1)	11.1 % (1)
1–3 years	37.5 % (9)	27.8 % (5)	33.3 % (3)
4–6 years	29.2 % (7)	27.8 % (5)	33.3 % (3)
7–9 years	12.5 % (3)	11.1 % (2)	11.1 % (1)
Over 10 years	12.5 % (3)	16.7 % (3)	11.1 % (1)
**Type of Contract**			
Fixed for 2 years or less	58.3 % (14)	66.7 % (12)	55.6 % (5)
Fixed for >2 years	8.3 % (2)	11.1 % (2)	11.1 % (1)
Open ended	8.3 % (2)	16.7 % (3)	0 % (0)
Permanent	25 % (6)	5.6 % (1)	33.3 % (3)
**Working Hours**			
Full-time	91.7 % (22)	94.4 % (17)	100 % (9)
Part-time	8.3 % (2)	5.6 % (1)	0 % (0)
**Salary**^c^			
£21,000–30,999	12.5 % (3)	11.1 % (2)	11.1 % (1)
£31,000–40,999	45.8 % (11)	44.4 % (8)	66.7 % (6)
£41,000–50,999	37.5 % (9)	38.9 % (7)	22.2 % (2)
>£51,000	4.2 % (1)	5.6 % (1)	0 % (0)
**CTUs Hybrid Working Policy**			
Min 60 %–80 % in office	25 % (6)	16.7 % (3)	22.2 % (2)
Min 20 %–40 % in office	12.5 % (3)	55.6 % (10)	44.4 % (4)
Flexible/Ad-hoc	25 % (6)	27.8 % (5)	33.3 % (3)
**Flexible Working Arrangement Agreed**			
Yes	45.8 % (11)	33.3 % (6)	22.2 % (2)
No	54.2 % (13)	66.7 % (12)	77.8 % (7)

a Three study advisory group (SAG) members also participated in the consensus panel survey, resulting in a total sample of n = 21, SAG characteristics are not included in this table.

b Six SAG members also participated in the consensus panel workshop, resulting in a total sample of n = 15, SAG characteristics are not included in this table.

c Salary was included as a broad proxy for level of seniority and role experience.

***Methods.*** Five focus groups were conducted and recorded online via videoconferencing software (Microsoft Teams), during standard working hours, and transcribed using the inbuilt transcription tool. Participants took part at their place of work, whether that was their home (hybrid working) or office. No written notes were taken during the focus groups. Focus groups were facilitated by an experienced qualitative female researcher (ER), with an MSc in Health Psychology, and specialism in qualitative research methods. ER was employed as a Research Fellow at the time of the study, she had experience of working within different UKCRC-registered units and had an interest in workplace counselling. ER was unknown to the study participants but had sent personal email invitations to them inviting their participation in the research. Participants were informed of the aim of the focus groups, which was to discuss and develop recommendations for the wellbeing challenges, related to flourishing, that we identified in our survey [36]. A topic guide was developed by the study team centred around promoting discussions and developing solutions to the challenges experienced by CTU staff which prevent flourishing, including, Growth (e.g., personal development), Environment (e.g., workload and work-life balance), Communication (e.g., with colleagues and seniors) and Acknowledgement (e.g., praise and recognition). The topic guide was reviewed by two individuals working within a CTU, who did not participate in the focus groups and were not part of the research team. Each focus group lasted for 90 min.

***Analysis****.* Focus group transcripts were reviewed for errors and coded using NVivo 12 software. It was not necessary to seek clarification from participants on the transcripts, and as such they were not contacted to review these. A deductive analysis approach was used to broadly code the data using key concepts included in a flourishing framework. An inductive approach was then used to explore for themes within these codes. Throughout the analysis a co-constructivist epistemology was taken by the two researchers (ER, SSH) who independently coded the data, with decisions reviewed and disagreements resolved through discussions. A co-constructivist approach was taken so that the recommendations generated were constructed through shared interactions, allowing insights to emerge dynamically as participants discussed and reflected on wellbeing challenges and potential solutions in their own contexts.

### 2.4 Stage 2: Consensus development

Consensus development consisted of two phases, a consensus panel survey, and a consensus panel workshop.

***Participants.*** The 24 participants, who attended the focus groups were invited to complete the consensus survey and join the consensus panel. Eighteen participants took part in a consensus survey, along with three members of our study advisory group (total n = 21, see Table 1). Nine participants took part in the subsequent consensus panel workshop, along with six members of our study advisory group (total n = 15, see Table 1), plus two researchers (ER, SSH).

***Methods.*** Recommendations for supporting staff to flourish in their roles were developed by the study team, drawing upon the findings of our previous national survey and focus group data. These recommendations were evaluated by the consensus development panel in the survey and panel meeting.

Due to the large number of items generated by the focus group analysis (see results), the survey was divided into four sections to reduce participant burden and disengagement. Items were rated to include, amend, or exclude. A free text option was provided so that further details could be obtained as to how the item should be amended if applicable. Items which had suggestions for improvement were modified based on this feedback prior to the meeting.

In the second phase, items which did not reach the pre-specified consensus criteria, or which were modified because of participant feedback, were discussed by the consensus panel members in an online workshop. Each item was briefly presented by a researcher after which the panel anonymously voted to ‘include’ or ‘exclude’ the recommendation using the polls function in Microsoft Teams. The researcher facilitated discussions but did not participate in voting. Items which reached consensus to include were also reviewed by the panel and suggestions for comprehension made where necessary.

***Consensus.*** Ratings in the consensus meeting were categorised as *Consensus to keep in the guidance:* ≥70 % agreement to include; *Consensus to not include in the guidance*: ≥70 % agreement to exclude. Where consensus was not reached, discussion followed among voters who had chosen to endorse a statement and voters that had not. Based on these discussions, final decisions were made as to whether to revise and include these statements or exclude them.

### 2.5 Stage 3: Guidance finalisation

Final wording for the guidance items along with the layout and structure were developed through an iterative review process between the study team and consensus meeting members through sharing draft versions.

## 3 Results

### 3.1 Participants

Participants’ characteristics are presented in Table 1. Across the three stages (focus groups, consensus panel survey, consensus panel workshops) participants were primarily white females. Most participants had been working in their CTU for at least 1 year and earned in the middle range salary bracket, which is a proxy for seniority and role experience. Those working in a trial management role were most represented, followed by those working in a senior leadership role and IT/data. Study Advisory Group (SAG) members also participated in the consensus panel, their details are not included in the participant characteristics table. All SAG members were white females, working on permanent/open-ended contracts. All bar two members had experience of working within a CTU in a senior role. The remaining two members included a Professor of Medical Education and Director of Equality, Diversity and Inclusivity (PH), and a Chartered Occupational Psychologist (LT).

### 3.2 Stage 1: Focus groups

Data codes and example quotes are presented in Table 2; these formed the basis of study team discussions to develop wellbeing recommendations, which were evaluated in the consensus stage of the project. No new major themes were evidenced after the fourth focus group.

**Table 2:** Framework analysis.

Primary Code	Secondary Codes	Focus Group Discussion Summary	Wellbeing Priority Item (developed by study team)
**Environment**	***Budgeting finances***	If more funding was allocated to a project, then it would be more likely to succeed, thus reducing Variations to Contracts (VTCs), burnout in staff, high turnover, research waste etc. Sometimes the issue is that the person responsible for the budget for a grant is so remote from the trial manager (TM) that they do not know what is going to be required in practical terms. Even if the budget has been put together well, the investigators, if they have never worked for a CTU before, view CTUs as really expensive, and start eroding the budget. It is important to empower CTU leads to engage with funders - to improve staff wellbeing by securing better resourced trials.	1. Budget development should evidence consideration of appropriate staff time allocation.
			2. Develop budgets for new grants with input from all relevant teams (e.g., data, IT, trial management) across the main job roles to ensure feasibility of the proposed resources.
			3. Resistance from investigators unfamiliar with CTUs towards cost allocation highlights the importance of making them aware of the role of the CTU and the significance of appropriately funded trials.
			4. Host knowledge-exchange workshops with study teams from different CTUs and funding bodies to better understand the budget development process and justification of resources.
			5. At end of study close-out procedures, reflect on actual staff and non-staff costs (including those sourced from other budgets) to inform future budgeting decisions.
			6. Where funding permits, core staff members (e.g., administrators, communication managers etc.) who are not trial bound, could be employed to work across trials and provide support easing some workloads.
			7. Reminder: Stand firm in your position when your experience indicates that a trial is underbudgeted. If collective pushback occurs, it could prevent the subsequent delivery of projects fraught with commonly occurring issues such as failure to meet milestones and staff turnover.
**Environment**	***Time and workload management***	Having dedicated time for planning at the beginning of the project would help with the management of the project and workload later on. Dedicated time for planning at the beginning of your day would also be useful - but there are often distractions like emails coming in. Some managers are ‘possessive’ of their trial, they see it as their baby, and so they do not delegate bigger tasks to trial assistants, coordinators etc. which can ultimately lead to burnout. A way around is to give your coordinators/assistants a task to do, and review it at the end. Be supportive, tell them you do not expect them to know what they are doing but to try it out. It gives them a chance to learn and get some experience, they are more able to provide support but also the control stays with the trial manager. Remind people - who may otherwise think that they are under time pressure and cannot possibly take on training someone on a task to be delegated - that by investing their time now, that means that that person can then relieve that pressure in the future. Rotate the tasks you delegate so the assistants get varied experience, and the managers get the support they need. It is a big jump between the trial coordinator and trial manager level. A trainee trial manager grade could help ease the workload of TMs and offer lower grade/level staff the opportunity to get valuable experience, so they are better able to climb the career ladder.	8. Set aside time at the beginning of the day/week to proactively shape your agenda, which can lead to increased productivity. Tip: Diarise weekly planning time; disable email/online teamwork platform notifications and avoid other distractions.
			9. Planning time at the beginning of a study as well as periodically during set-up and recruitment should be taken into account in grant planning. More time can aid in identifying and mitigating risks reducing workload later on.
			10. Consider introducing a trainee manager role or other apprenticeship training to improve mobility within job roles/pay grades and reduce staff pressures.
			11. Delegation: Be mindful of task hoarding. Letting tasks pile up on your to-do list(?) without effectively delegating to others can cause delays and could even contribute to burnout. Delegation should evidence reflection on task urgency, staff capacity, role expectations and skill development. Tips: Engage with training/resources on effective delegation, prioritisation, and productivity. - Use project management software for task tracking and team collaboration. - Assign new tasks to coordinators/assistants and review them upon completion. Be clear from the start that you do not expect them to know all the answers. - Rotate task delegation to enhance team skill-base.
**Environment**	***Flexible working***	Staff allocated to backup trials can help with any tasks that urgently need to be done at the office while other staff are working from home. Staff should be each other's ‘backup’ so when they are in turn working from home, their colleagues can help with anything urgent needed at the office. Overtime done at the beginning of the week cannot be exchanged with e.g., a doctor's appointment or leaving early on a Friday in some workplaces. (Not referring to Time off in Lieu (TOIL) in lower grades but for higher grades who cannott incur TOIL). If there was more flexibility with this, that would also be a way to show people who are working hard, that they are valued. The four-day working week, is difficult to implement. Some Universities are considering it but may take a while to implement. It is the one change that would make the biggest difference for the biggest number of people.	12. Where tasks require urgent attention in the office, establish an agreed staff rota, while respecting flexible arrangements. Staff working in the office can collectively manage urgent administrative tasks on days when other colleagues are working remotely. Thus, promoting equality in hybrid working.
			13. Consider adopting a flexi-time arrangement; a practice often enforced in the public sector to give staff greater autonomy in managing their time – linked to greater job satisfaction. With flexitime, staff can be allowed to work flexibly and reclaim up to two days of TOIL per month.
			14. Consider the merit of implementing a four-day working week. Staff feedback indicates that this change would bring a significant positive impact on most CTU staff's wellbeing. Any such changes require engaging with the relevant institution leads and should only be adopted when in line with organisational policy.
**Environment**	***Meetings***	Meetings should minimise chit-chat, make sure there is a plan and clear actions at the end. Consider rotating chairs, such as in staff briefings, so everyone gets experience and learns from each other, and they feel acknowledged and involved.	15. If you need to communicate information, consider the most efficient method of delivery (email, message, meeting etc.) and who actually needs to be included.
			16. Focus meetings around objectives with a clear agenda and action points. Tip: Rotate meeting Chairs (e.g., in Trial Managers' meetings) so all managers practice effective meeting management.
**Environment**	***Meaningful work***	The CTU should share the impact that previously completed trials have made, in an informal group meeting format where there is opportunity for discussion, perhaps with new starters or on a regular basis. A lot of the work that has been done is forgotten. It would be nice to see the body of evidence you are building/adding to.	17. Contributing to impactful research is important to career satisfaction in CTU staff. Periodically share impact reflections/stories on completed trials. Tip: Dissemination can be made via group meetings, key results/an impact statement can also be circulated via email.
**Environment**	***Work-life balance***	Line Manager (LM) discussions with their line reports: there should be flexibility with TOIL across the board but emphasise that TOIL should not be taken to the detriment of annual leave. Taking an afternoon back is not the same as switching off. Having had work experience at CTUs without a TOIL practice enforced, the difference was noteworthy. It is helpful to have this practice in a structured way followed by the whole CTU. Then you would not feel guilty about asking - the time would be available for you to use when needed. Important to have some LM time to focus on wellbeing.	18. Working overtime should be monitored. Consider allowing greater flexibility in taking Time Off In Lieu (TOIL) of additional hours worked, regardless of staff grade/level. Emphasise to staff that TOIL does not replace annual leave in terms of benefits to wellbeing.
**Communication**	***Transparency***	A rep from each grade/level to go to senior meetings [there should be dedicated time during those for this purpose], raise points and feedback to their grade. Different teams to be consulted in the decision process for changes that may affect them. For example, if the CRF structure/format changes, it should not only be a Data team job that is communicated to the rest of the CTU but those in Trial management should be made part of the updating process. This would save a lot of time and issues later on.	19. Brief updates from CTU team representatives could be included in Trial Managers' meetings or CTU staff briefings so everyone is kept updated.
			20. Invite a spokesperson to represent the voice of all team members across grades/levels at senior management/operations meetings.
			21. Key changes in Standard Operating Procedures (SOPs) (e.g., significant modifications of CRF forms) should be briefly discussed across CTU teams to ease implementation and ensure everyone feels part of the process.
**Communication**	***Communication with line managers and seniors***	CTUs should be advised that they have a responsibility about their employees' line management even when the line manager is the Chief Investigator (CI). There should be enough people at each level including Senior Trial Management (STM) level to support staff - as when the CI is the line manager (instead of an STM) line reports may not be able to ask for advice and get the support needed. The less hierarchical a CTU is, the better the communication lines are.	22. Fostering strong rapport with senior trial managers could ensure that you are well-informed of any higher-level issues, to provide the necessary support to prompt resolution.
			23. Retain oversight over employees who are line managed by Chief Investigators (CIs).
			24. Invite staff of different specialties/levels to shadow senior colleagues during early meetings with CIs; this could bolster their professional growth and reinforce a culture of respect, contributing to positive CI-trial team relationships.
**Communication**	***Specific ways of working***	Working in smaller teams can be helpful because closer contact with line managers can reduce communication issues. Staff should be available for queries on Teams. There should not be an unnecessary amount of emails for things that in the past you would call or talk to someone. It can be important to protect time for work without distractions. Some staff may perceive Teams as a friendly informal chat and cross professional boundaries, which can result in communication issues. Having lunch breaks is really important. They can reenergise staff and can enhance productivity. Lunchbreak with colleagues - can make the workplace a nicer place to work at - it helps everyone work more efficiently. Support from the line manager in adopting this habit could reduce guilt and encourage uptake.	25. If your team is large, consider establishing sub-groups to promote clear communication.
			26. Develop etiquette guidance for online teamwork platforms, such as Microsoft Teams or Zoom, including appropriateness, frequency of messaging, availability (e.g., respecting protected time, lunchtime etc) and content (i.e., what should be communicated via email vs Teams).
			27. Communication tools should be developed to ascertain if individuals are available to offer short-term/temporary support to over-stretched studies during high pressure time points. Tips: Coordinators/Assistants could provide backup on another trial in addition to the one(s) they are working on. They could be kept updated (e.g., via meeting minutes) and could then provide support at high-pressure times (e.g., to cover trial staff leave). - Develop and maintain a handover template per study for absences and turnover. Allow sufficient time for developing and updating this template.
			28. Encourage staff to have lunch breaks away from their screen whether working remotely or in person. Vocalising your support as a manager could reduce feelings of guilt in staff for not being immediately available.
**Communication**	***Inter-team working***	Team leaders, such as from Stats, TM etc. could meet once a month or more frequently, depending on the CTU's size, to update each other on projects. This helps to know what is coming, how busy people are, enable clear communication and improve workload management across teams. We should consider, for example, weekly admin meetings where everyone's to-do is shared with colleagues, any pressures are identified, including upcoming ones, and that is communicated to 'backups' and managers as needed so they can reallocate resources. Some CTUs may have a single Quality Assurance (QA) officer, researcher etc., who may not be included in TM or other group meetings so finding ways to make them feel part of the team is important. Team-building days - can be cheesy but if done well, can be invaluable. Activities can be games - there are companies that specialise in that. Not everyone is available or likes going out for a drink or meal while a team-building activity can work for everyone.	29. CTU team leaders (e.g., Programming, Statistics) should meet periodically to update each other on trial progresses and challenges (on-going or upcoming) to facilitate clear communication on team capacity and brainstorm solutions.
			30. Consider organising brief agile meetings within trial teams so tasks to be prioritised and weekly targets are agreed to promote efficient use of time.
			31. Be aware that integration within the CTU may be harder for employees working outside established teams (e.g., a quality assurance officer). Be mindful to be inclusive in CTU activities/relevant communications.
			32. In-person and/or remote team building activities should be supported at regular intervals by CTUs to improve social wellbeing. Organise different activities to maximise inclusion. Drinks or a meal after work are not activities accessible to all.
			33. Participant feedback has indicated that team lunches may also improve team communication, these do not need to be in person and should be optional. 34. CTU facilitated yoga/mindfulness/breathing work could be implemented as an optional weekly lunchtime activity. Relevant resources can be accessed online e.g., through university wellbeing webpages.
**Communication**	***Inter-team working***	Check to see what University resources are available for wellbeing activities that the CTU may be eligible for. Team building activities of different sorts can take place within the CTU out of work hours – particularly helpful if CTU has limited/no funding for external events. Without any pressure to attend every time. They could help build better communication and break down barriers that may have been there before.	35. Apply for university funding where available for wellbeing activities.
			36. Consider organising refreshment breaks, including protected time and provision of the refreshments, where possible, to promote a positive team environment and encourage breaks.
**Communication**	***Role expectations***	Understanding people's roles, and when they should be brought in, could empower everyone and ensure continuity, for example, if a manager is off sick. It could also ease workloads - the trial manager does not always have to be the in-between to organise meetings such as between Data & Stats, and it also helps coordinators who want to become trial managers to be more involved, ease the trial managers' workload but also learn and grow.	37. Raise awareness about the roles and staff members holding these positions through activities. For example, ‘A day in the life of a data coordinator’ to promote role awareness: - Include details about their career paths to highlight diversity in backgrounds. - Invite external collaborators, such as a Health Economics team based in a collaborating institution, to take part.
			38. Promote an understanding of the dual roles (academic and project management) of CTU staff, as well as associated responsibilities, to cultivate more successful collaborations.
**Communication**	***Induction***	CTU specific induction including up-to-date chart with all team members and what they do would be particularly helpful with remote or hybrid working. New starters could have a quick Teams meeting with all leads, like Data, IT, Nursing, Heads of CTUs, so they are introduced to them and feel more comfortable to reach out to them if and when needed.	39. A CTU-specific induction should be conducted; consider a live (regularly updated) organogram with names and job titles to improve integration of new team members.
			40. Organise brief introductions to all team leads within the CTU – online introductions are likely to be welcomed and save time.
			41. Record videos with an overview of each CTU team's main responsibilities and ensure everyone understands how the different teams fit together.
***Growth***	***Protected time for training***	No-meeting initiatives are being trialled at multiple CTUs. They are not always respected by external collaborators, but they are respected internally and have reduced the number of meetings. Employees now have more headspace for trial planning, training, other career growth activities. There is a need to start small and be flexible with when/how this time is taken so it is adopted by everyone across all grades/levels. Things like a directory of experts in different skills at the CTU could help spread out supporting work across many staff members. Peer-to-peer support could help spread the burden and it can even become a formal responsibility as part of people's job roles. Seniors need to be supportive and encouraging of staff blocking time to give a talk or seminar on something they are experienced in and sharing that knowledge with the rest of the CTU. We need mentors for CTU staff, some CTUs seem to offer more academic or research-centric mentorship programmes which are not available to CTU staff. In general, helpful to have someone else to lean on for support outside your line manager. Mentoring helps with sharing of expertise and development but also helps build better relationships. Time spent to help colleagues is not protected. Some CTUs ask their staff to complete timesheets and there is no option to allocate time for helping/miscellaneous work other than budgeted trials. People say things like ‘they should have asked their TM’ or ‘they should have done this or that’ which make staff hesitate to help.	42. Consider introducing a no-meeting time initiative when training and career development is encouraged. Allow for flexibility in how/when this time is taken (e.g., opt for a monthly minimum quota rather than a fixed day).
			43. Initiatives such as CTU-wide protected no-meeting time should be respected and honoured across all levels/grades. Tips: Use this time to attend training or complete other career development activities. - Support the inclusion of protected no-meeting time in staff signatures and online diaries to promote wider awareness.
	***Sharing of expertise***		44. Create and maintain a directory of CTU staff expertise (i.e., names and skill(s) they are experienced in) so colleagues can approach experts directly.
			45. Peer-to-peer support (i.e., a buddy system) could help knowledge exchange and career development.
			46. Experienced staff members (on a rota) to host drop-in sessions that colleagues can access to have their queries resolved.
			47. Support and encourage staff across all levels to block time to give a talk on a topic they are experienced in to share their learning.
			48. Create a Microsoft Teams channel/other online teamwork platform community where queries can be posted and answered by colleagues.
			49. Host lunchtime seminars to cover a variety of topics, such as examples of successful recruitment strategies, SAE handling etc., to facilitate sharing of expertise in a hybrid working environment.
			50. Establish a CTU-specific mentorship scheme. Participant feedback has indicated that equivalent research/academic programmes do not fully capture the responsibilities and career trajectory of CTU staff and in some cases are not even available to CTUs.
			51. Establish a CTU-based reverse mentorship programme to support new line managers as well as senior leaders, who may benefit from being exposed to different employee backgrounds and experiences. Ensure mentors-mentees do not belong to the same CTU team.
			52. Where staff are required to complete timesheets, they could be allowed to put down any time spent helping colleagues as trial work. If all staff follow this practice, helping time would be evenly spread across trials.
**Growth**	***New starters***	Different arrangements may be useful for new starters who are recent graduates – especially if they do not have any or have limited work experience – they need more support.	53. Where other strategies have been unsuccessful, consider encouraging new employees who are recent graduates to temporarily spend more time in the office, so they are more exposed to established ways of working and code of conduct. Adopt an individualised approach based on people's needs and learning styles.
**Growth**	***Specific training***	Training to focus on other aspects of work such as wellbeing, project management, giving and receiving feedback etc. should be encouraged, not just skills for clinical trials. Things need to be in one place and easy to find. One place should be created, where CTU staff can go and see what kind of events are happening this month. A website, newsletter and social media accounts would also be helpful. It is not always possible for everyone to go to all training. Where not possible for people to attend training, one/a few volunteers can go and then report back to the wider team - which can be recorded for anyone to listen who may not be able to attend. People get promoted to senior roles often based on their skills such as research but eventually find themselves managing people - and the assumption is being made that they know how to do that. Line manager or relevant training should be in place by the institution and where itis not, it should be strongly encouraged by the CTU across the board from new managers up to directors. This may help to bring everybody closer together. LM training on active listening skills, managing different personalities etc. would be helpful but it should be short (e.g., 20 min) and accessible online. That way it can be easily done when needed, e.g., before having a difficult conversation with someone. There is so much information that TMs have to keep in their heads, we should not expect them to do it once and just hold that in their heads too.	54. Staff should attend training on all aspects of work including (but not limited to), giving feedback, project management, mental health, and wellbeing, as well as technical/task-speVcific skill training. - Consider attending mental health training; it can increase self-awareness, understanding of others and improve team performance.
			55. Create and maintain a centralised resource/directory with all training workshops/courses available. Tips: This could be a Teams channel or a SharePoint spreadsheet that staff can post on and find available training opportunities. - Where attendance at training is not feasible for all, designated volunteers (on a rota) can attend and feed back to the wider team on key learnings. - Ask staff to log brief feedback and attendance at training in a centralised resource to aid colleagues in deciding whether a session would be beneficial to attend.
			56. Introduce practical cross-disciplinary training exercises at group meetings. Tips: Staff on a rota could present a study and ask colleagues to identify challenges/solutions. The study could be a new grant, thus using this as an opportunity to problem-solve as a team, or a published clinical trial. Include a multiple-choice anonymous polling for maximum learning benefits and to reduce evaluation anxiety. - Similarly ask colleagues to solve a problem/achieve a task using the process outlined in a SOP. Consider who should be involved, when and how.
			57. Encourage line managers across all levels/grades to attend line managers'/leadership training.
			58. Where the CTU leads the development of line management training consider the following: - Participant feedback indicates that staff connect with and retain information that included stories/experiences that they can relate to. - Include content such as active listening skills, managing different personalities etc. - Break training into separate modules <20 min; so, staff can refer back as and when needed without having to sit through a long module. - Training should be recorded and online options offered for ease of access.
**Growth**	***Protecting training resources***	CTU staff are often considered professional services and therefore do not have access to funding for training available to researchers. Additional justifications as to why the training is necessary should not be required.	59. Where line management training is offered by the University but not available to CTU staff, consider engaging with the relevant University leads to request access is granted. Emphasise how integral it is for career development at CTUs.
**Acknowledgement**	***Showing appreciation***	It does not have to be constant praise or anything extravagant but small things to show genuine appreciation can go a long way. Teams has an in-built praise tool that can be used for that. We need to increase respect - everyone feeling like they are part of a big effort. Focus on the positive things too - recognise & appreciate the team - instead of only focusing on what has not been done/or is next due. Trials take a long period of time and that often translates to a change in staff. A registry or some sort of central tracking of staff would ensure that even when staff have left, they are acknowledged in trial publications. Raise awareness about the importance of recognition at all levels. Some people think it is needy or that adults are not supposed to need to feel appreciated, that is something you do for children they say, but showing appreciation to people whose hard work will not be recognised another way – like a promotion - is really important. It is important to be self-aware; look at your work and acknowledge all the good things you have done. Keep a log of them to raise them at your annual development meeting. It is also important for you to recognise what you are doing well and not just beat yourself up for all the things that have gone wrong, or you have not done to your or others' standards. The positive act of appreciating yourself may also help you notice other things that others are doing well; and more likely to say so. The School does not know what the CTU does. The CTU is not listened to and the structure of the School and CTU means that the CTU is not able to access funding for things like training. “My dream would be that the university would recognise our role as not being just 100 % grant funded. We work on grants, but also that there are structural aspects that we have to do, basically in our spare time, and to get that sort of protected time to some extent, or at least recognition within the wider university to implement things like this, yeah, that that would help my wellbeing.”	60. AdopVt small ways to show appreciation such as: - A little ‘thank you’ goes a long way. - Call/talk to your managee after a big meeting and compliment them on a job well done. - If you are using an online platform such as Microsoft Teams, use the built-in Praise tool to thank your colleagues. -Include the voice of all team members (Data, IT, Administrators) in a TMG agenda to show appreciation/recognition for their contributions. - Allocate some CTU staff briefing time to recognise trial successes and appreciate team members who have contributed to these across all levels/grades. - Team-building activities can be organised as recognition for hard work. 61. Feedback should be constructive. Start with praise for managees' accomplishments before giving feedback on areas needing improvement. Feedback should always be balanced, timely and include examples. Attend a course or access relevant resources on giving and receiving feedback.
			62. Nominate colleagues through the University reward scheme where one is available. Ensure that colleagues of all specialties are recognised for their contributions. If a university reward scheme does not exist, consider setting up a Unit-level one.
			63. Publication policies should be acknowledging trial management staff on par with other roles.
			64. Taking the time to recognise your efforts can train you to notice colleagues' efforts too and overcome our tendency to take people we work closely with for granted. By appreciating your efforts and those of your colleagues, you will be contributing to a circle of positivity where appreciation becomes a natural part of how your CTU operates.
			65. Give yourself credit for your own work. Do not just focus on mistakes or where you have performed below yours or others' standards. Keep track of your accomplishments and raise them at your appraisal.
			66. Maintain a log with the names of all staff who have worked on a trial to ensure they are acknowledged in relevant publications.
			67. Engage with relevant University leads to raise awareness of the role of the CTU, its structure and needs as well as its contribution to university research and funding. Highlight the importance of CTU staff having equal access to funding, training etc. as their university colleagues. Liaise with other CTUs' leads who have successfully engaged in such discussions to learn from their experience.

Four major themes were evident: environment, communication, growth, and acknowledgement. These and their sub-themes are reported below.

The broader working *environment* was considered in relation to wellbeing, with recommendations highlighting the need for improved resource management, especially staff time and workload, from project planning to completion. Effective flexible working was emphasised, with strategies to ensure equality for administrative roles that may require more office presence. Time Off In Lieu (TOIL), flexi-time and other such arrangements were seen as ideal for boosting autonomy (intrapersonal flourishing) and maintaining good work-life balance. The strategies discussed included running productive meetings and better informing trial staff about the impact and implementation of their work to foster a sense of meaningful engagement; linked to higher flourishing.

Discussions highlighted the importance of effective *communication* within a CTU. Key topics included improving induction content, increasing awareness of different role responsibilities across a CTU and setting clear role expectations. Emphasis was placed on promoting good inter-team communication and engaging effectively with seniors. Strategies were discussed to help employees feel supported and integrated into larger and wider teams, such as creating sub-groups to disseminate information. Additional topics included developing online communication etiquette and promoting transparent and inclusive decision-making.

Participants also discussed strategies to promote employee *growth*, such as sharing expertise within a multi-disciplinary CTU. They emphasised the importance of peer-to-peer support and drop-ins. In-person support was highlighted as especially beneficial for new starters at the early stages of their careers. The need for wider skills training, including leadership development and core trial management, was noted. Ensuring CTU staff have equal access to these training opportunities, comparable to other employees within their institution, was deemed crucial, but not always realised. Finally, creating a culture which supports growth within the CTU was valued. This could be achieved through protected no-meeting time initiatives enabling employees to pursue training and other development opportunities.

Participants discussed the need for embedding *acknowledgement* strategies within a CTU, emphasising the importance of recognising individual contributions in large teams. These strategies could range from simple thank you notes to more tangible rewards, such as those available through institutional initiatives such as reward schemes or staff awards. Participants noted that individuals should also take responsibility for recognising their own achievements to foster a culture of positive recognition at all levels. Furthermore, appropriately recognising the contributions of the wider team in accordance with publication policies was highlighted as important. Finally, recognising the contributions, in terms of research impact and funding generation, of the CTU within their host organisation was covered. Recognition of this could give staff the opportunity to access funding and resources not currently available to them.

### 3.3 Stage 2: Consensus development

**Fig. 1:**
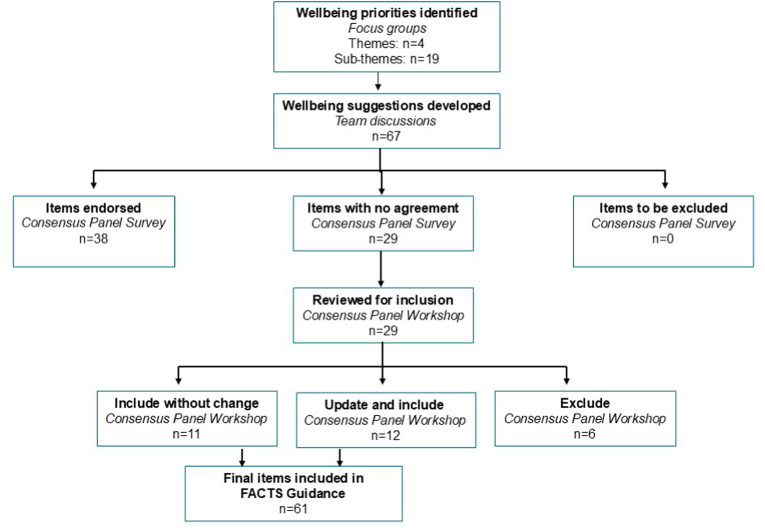
FACTS item development process.

The guidance development process is illustrated in Fig. 1.

Sixty-seven recommendations (Table 2) for supporting staff to flourish in their roles were developed by the study team, drawing upon the findings of our previous national survey [36] and the focus group data.

In the first phase of consensus development the consensus survey (n = 18 participants, n = 3 study advisory members) reached agreement to include 38 items, no consensus was reached for 29 items, and no items reached agreement to exclude (Table 3). The 29 items were revised by the study team based on free-text comments and the focus group data before being further evaluated in the consensus workshop.

**Table 3:** Consensus panel survey and workshop outcome.

Wellbeing Item	Consensus Survey				Wellbeing Item Revised	Consensus Workshop		
	Include	Amend	Exclude	Decision		Include	Decision	Final wording
**ACKNOWLEDGEMENT**								
Give yourself credit for your own work. Do not just focus on mistakes or where you have performed below yours or others' standards. Keep track of your accomplishments and raise them at your appraisal.	50.0 %	50.0 %	0.0 %	No consensus	Don't be afraid to give yourself credit for your own work. Do not just focus on mistakes or where you feel you have performed below yours or others' standards. Keep track of your accomplishments and learning achievements to raise them at your appraisal.	90 %	Update and include	Don't be afraid to give yourself credit for your own work. Do not just focus on mistakes or where you feel you have performed below yours or others' standards. Record your accomplishments and learning achievements to raise them at your appraisal.
Taking the time to recognise your efforts can train you to notice colleagues' efforts too and overcome our tendency to take people we work closely with for granted. By appreciating your efforts and those of your colleagues, you will be contributing to a circle of positivity where appreciation becomes a natural part of how your CTU operates.	75.0 %	25.0 %	0.0 %	Include	N/A	N/A	Include without change	No change from original
Feedback should be constructive. Start with praise for managees' accomplishments before giving feedback on areas needing improvement. Feedback should always be balanced, timely and include examples. Attend a course or access relevant resources on giving and receiving feedback.	75.0 %	25.0 %	0.0 %	Include	N/A	N/A	Include without change	No change from original
Nominate colleagues through the University reward scheme where one is available. Ensure that colleagues of all specialties are recognised for their contributions. If a university reward scheme does not exist, consider setting up a Unit-level one.	50.0 %	50.0 %	0.0 %	No consensus	Nominate colleagues through your organisation's reward scheme where one is available. Ensure that colleagues of all specialties are recognised for their contributions. If a reward scheme does not exist, consider setting up a Unit-level one.	100 %	Include without change	No change from first revision
Adopt small ways to show appreciation such as: A little ‘thank you’ goes a long way. - Call/talk to your managee after a big meeting and compliment them on a job well done. - If you are using an online platform such as Microsoft Teams, use the built-in Praise tool to thank your colleagues. - 1 the voice of all team members (Data, IT, Administrators) in a TMG agenda to show appreciation/recognition for their contributions. - Allocate some CTU staff briefing time to recognise trial successes and appreciate team members who have contributed to these across all levels/grades. - Team-building activities can be organised as recognition for hard work.	62.5 %	37.5 %	0.0 %	No consensus	Adopt small ways to show appreciation such as: - Saying ‘thank you’ goes a long way. - Call/talk to your managee after meetings and compliment them on a job well done. - If you use an online collaboration platform, make use of the built-in Praise tools. - Include the voice of all team members in a TMG agenda to recognise their contributions. - Allocate CTU staff meeting time to recognise trial successes and all team members' contributions. - Organise team activities to recognise hard work.	93 %	Update and include	Adopt small ways to show appreciation such as: - Saying ‘thank you’. It goes a long way. - Call/talk to your line reports after meetings and compliment them on a job well done. - If you use an online collaboration platform, make use of the built-in Praise tools to formally acknowledge your colleagues' hard work. - Include the voice of all team members in a TMG agenda to recognise their contributions. - Allocate CTU staff meeting time to recognise trial successes and all team members' contributions. - Organise team activities to recognise hard work.
Publication policies should be acknowledging trial management staff on par with other roles.	62.5 %	37.5 %	0.0 %	No consensus	Introduce publication policies which fairly reflect all clinical trial staff's level of contributions.	100 %	Update and include	Introduce publication policies which fairly reflect all clinical trial staff's level of contributions.
Maintain a log with the names of all staff who have worked on a trial to ensure they are acknowledged in relevant publications.	87.5 %	12.0 %	0.0 %	Include	N/A	N/A	Include without change	No change from original
Engage with relevant University leads to raise awareness of the role of the CTU, its structure and needs as well as its contribution to university research and funding. Highlight the importance of CTU staff having equal access to funding, training etc. as their university colleagues. Liaise with other CTUs' leads who have successfully engaged in such discussions to learn from their experience.	37.5 %	63.0 %	0.0 %	No consensus	Engage with senior organisational leads to raise awareness of the important contributions of a CTU and the need for trial staff to have equal access to funding, training etc. as their university/Trust colleagues. Share your knowledge and experience of this process with other CTU leads.	100 %	Include without change	No change from first revision
**COMMUNICATION**								
If your team is large, consider establishing sub-groups to promote clear communication.	75.0 %	12.5 %	12.5 %	Include	N/A	N/A	Include without change	No change from original
Be aware that integration within the CTU may be harder for employees working outside established teams (e.g., a quality assurance officer). Be mindful to be inclusive in CTU activities/relevant comms.	62.5 %	37.5 %	0.0 %	No consensus	Integration within the CTU may be harder for employees working alone or in smaller teams. Be inclusive in CTU activities/relevant communications.	100 %	Include without change	No change from first revision
Develop etiquette guidance for online teamwork platforms, such as Microsoft Teams or Zoom, including appropriateness, frequency of messaging, availability (e.g., respecting protected time, lunchtime etc) and content (i.e., what should be communicated via email vs Teams).	100.0 %	0.0 %	0.0 %	Include	N/A	N/A	Include without change	No change from original
CTU team leaders (e.g., Programming, Statistics) should meet periodically to update each other on trial progresses and challenges (on-going or upcoming) to facilitate clear communication on team capacity and brainstorm solutions.	62.5 %	37.5 %	0.0 %	No consensus	CTU team leaders to share current and forecasted capacity within their team, updates on trial progresses, challenges and solutions.	87 %	Update and include	CTU team leaders to update each other on current and forecasted capacity within their team, trial progresses, challenges and solutions.
Brief updates from CTU team representatives could be included in Trial Managers' meetings or CTU staff briefings so everyone is kept updated.	62.5 %	37.5 %	0.0 %	No consensus	Brief updates from CTU team representatives could be included in staff meetings so everyone is kept updated.	80 %	Update and include	Updates from team representatives could include current challenges faced so colleagues are informed/can contribute to issue resolution.
Key changes in Standard Operating Procedures (SOPs) (e.g., significant modifications of CRF forms) should be briefly discussed across CTU teams to ease implementation and ensure everyone feels part of the process.	75.0 %	12.5 %	12.5 %	Include	N/A	N/A	Update and include	Feedback should be sought from CTU teams prior to key changes in Standard Operating Procedures (SOPs) (e.g., significant modifications of CRF forms) being implemented to ensure issues are considered from different perspectives and everyone feels part of the process.
Consider organising brief agile meetings within trial teams so tasks to be prioritised and weekly targets are agreed to promote efficient use of time.	100.0 %	0.0 %	0.0 %	Include	N/A	N/A	Include without change	No change from original
Raise awareness about the roles and staff members holding these positions through activities such as ‘A day in the life of a data coordinator’ to promote role awareness: - Include details about their career paths to highlight diversity in backgrounds. - Invite external collaborators, such as a Health Economics team based in a collaborating institution, to take part.	75.0 %	25.0 %	0.0 %	Include	N/A	N/A	Include without change	No change from original
Invite a spokesperson to represent the voice of all team members across grades/levels at senior management/operations meetings.	62.5 %	25.0 %	12.5 %	No consensus	Invite a spokesperson to represent the voice of staff from all grades/levels at senior management/operations meetings.	93 %	Update and include	Ensure the voice of staff from all grades/levels is heard at senior management/operations meetings. For example, consider inviting a different staff spokesperson to these meetings periodically.
Fostering strong rapport with senior trial managers could ensure that you are well-informed of any higher-level issues, to provide the necessary support to prompt resolution.	37.5 %	37.5 %	25.0 %	No consensus	Actively listen to any issues CTU team leads raise and action any changes promptly to foster trust and rapport with them. This could ensure that you are well-informed of any higher-level issues they face promptly and could provide the necessary support to lead to resolution.	55 %	Exclude	N/A
A CTU-specific induction should be conducted; consider a live (regularly updated) organogram with names and job titles to improve integration of new team members.	87.5 %	12.5 %	0.0 %	Include	N/A	N/A	Update and include	A CTU-specific induction should be conducted. 1. Consider creating and maintaining a live organogram with names and job titles to improve integration of new team members. 2. Organise brief introductions to all team leads within the CTU – online introductions are likely to be welcomed and save time.
Organise brief introductions to all team leads within the CTU – online introductions are likely to be welcomed and save time.	62.5 %	25.0 %	12.5 %	No consensus	Organise brief introductions to all team leads within the CTU - consider hosting these online to save time.	93 %	Include without change	No change from first revision
Record videos with an overview of each CTU team's main responsibilities and ensure everyone understands how the different teams fit together.	37.5 %	37.5 %	25.0 %	No consensus	Provide an overview of each CTU team's main responsibilities and ensure everyone understands how the different teams fit together.	100 %	Include without change	No change from first revision
Retain oversight over employees who are line managed by Chief Investigators (CIs).	71.4 %	28.6 %	0.0 %	Include	N/A	N/A	Update and include	Retain oversight over employees who are line managed by Chief Investigators (CIs) to ensure CTU processes are followed and they receive the same level of support as CTU-managed staff.
Promote an understanding of the dual roles (academic and project management) of CTU staff, as well as associated responsibilities, to cultivate more successful collaborations.	100.0 %	0.0 %	0.0 %	Include	N/A	N/A	Update and include	Promote an understanding of the dual roles (academic and project management) including the associated responsibilities and knowledge bank, to cultivate more successful collaborations.
Invite staff of different specialties/levels to shadow senior colleagues during early meetings with CIs; this could bolster their professional growth and reinforce a culture of respect, contributing to positive CI-trial team relationships.	57.0 %	43.0 %	0.0 %	No consensus	Invite staff of different specialties and grades/levels to shadow senior colleagues during early meetings with CIs. This could contribute to professional growth and a culture where staff are respected across levels/grades.	93 %	Update and include	Invite staff of different specialties and grades/levels to accompany senior colleagues during early meetings with CIs. This could contribute to professional growth and a culture where staff are respected across levels/grades.
Communication tools should be developed to ascertain if individuals are available to offer short-term/temporary support to over-stretched studies during high pressure time points. Tips: Coordinators/Assistants could provide backup on another trial in addition to the one(s) they are working on. They could be kept updated (e.g., via meeting minutes) and could then provide support at high-pressure times (e.g., to cover trial staff leave). - Develop and maintain a handover template per study for absences and turnover. Allow sufficient time for developing and updating this template.	57.0 %	28.5 %	14.5 %	No consensus	Develop a system so individuals can offer short-term/temporary support to over-stretched studies during high pressure time points. Tips: - Try to forecast busy times when staff are needed. Keep staff updated with the minimally important information so they are able to provide cover as needed. - Where budget allows, consider using temporary staffing services/Unitemps. - Keep a clear up-to-date action priority list to be used during absences and turnover.	100 %	Include without change	No changes from first revision
Social wellbeing in person and remote team building activities should be supported at regular intervals by CTUs to improve social wellbeing. Organise different activities to maximise inclusion. Drinks or a meal after work are not activities accessible to all.	85.7 %	14.3 %	0.0 %	Include	N/A	N/A	Update and include	In- person and/or remote team building activities should be supported at regular intervals by CTUs to improve social wellbeing. Organise different activities to maximise inclusion. Drinks or a meal after work late in the evening are not activities accessible to all.
Encourage staff to have lunch breaks away from the screen whether working remotely or in person. Vocalising your support as a manager could reduce feelings of guilt in staff for not being immediately available.	85.7 %	14.3 %	0.0 %	Include	N/A	N/A	Update and include	Encourage staff to have lunch breaks away from their desks whether working remotely or in person. Being supportive as a manager could reduce feelings of guilt in staff for not being immediately available.
Participant feedback has indicated that team lunches may also improve team communication, these do not need to be in person and should be optional.	57.1 %	14.3 %	28.6 %	No consensus	No changes.	47 %	Exclude	N/A
CTU facilitated yoga/mindfulness/breathing work could be implemented as an optional weekly lunchtime activity. Relevant resources can be accessed online e.g., through university wellbeing webpages.	71.4 %	14.3 %	14.3 %	Include	N/A	N/A	Include without change	No change from original
Apply for university funding where available for wellbeing activities.	85.7 %	0.0 %	14.3 %	Include	N/A	N/A	Include without change	No change from original
Consider organising refreshment breaks, including protected time and provision of the refreshments, where possible, to promote a positive team environment and encourage breaks.	71.4 %	14.3 %	14.3 %	Include	N/A	N/A	Include without change	No change from original
**ENVIRONMENT**								
Consider introducing a trainee manager role or other apprenticeship training to improve mobility within job roles/pay grades and reduce staff pressures.	57.0 %	43.0 %	0.0 %	No consensus	Consider introducing a trainee role with apprenticeship-style or “on the job” training to increase skill development, facilitating mobility within job roles/pay grades and reducing staff pressures.	53 %	Update and include	Consider introducing a trainee role with apprenticeship-style or “on the job” training to increase skill development, facilitating mobility within job roles/pay grades and reducing staff pressures. Trainee manager roles have been piloted in different CTUs with positive feedback.
Be mindful of task hoarding. Letting tasks pile up on your to-do without effectively delegating to others can cause delays and could even contribute to burnout. Delegation should evidence reflection on task urgency, staff capacity, role expectations and skill development. Tips: Engage with training/resources on effective delegation, prioritisation, and productivity. - Use project-management software for task tracking and team collaboration. - Assign new tasks to coordinators/assistants and review them upon completion. Be clear from the start that you do not expect them to know all the answers. - Rotate task delegation to enhance team skill-base.	86.0 %	14.0 %	0.0 %	Include	N/A	N/A	Update and include	Be mindful of task hoarding. Letting tasks pile up on your to-do list without effectively delegating to others can cause delays and could even contribute to burnout. Delegation should evidence reflection on task urgency, staff capacity, role expectations and skill development.
Set aside time at the beginning of the day/week to proactively shape your agenda, which can lead to increased productivity. Tip: Diarise weekly planning time; disable email/online teamwork platform notifications and avoid other distractions.	71.4 %	0.0 %	28.6 %	Include	N/A	N/A	Include without change	No change from original
Planning time at the beginning of a study as well as periodically during set-up and recruitment should be taken into account in grant planning. More time can aid in identifying and mitigating risks reducing workload later on.	43.0 %	43.0 %	14.0 %	No consensus	Strategic planning time at the beginning of a study as well as strategic review periods up until the end of recruitment should be taken into account at the grant application stage and where possible incorporated in the GANTT. More planning time can aid in identifying and mitigating risks reducing reactive problem-solving and associated heavy workload.	79 %	Update and include	When developing a project GANTT chart, include time for strategic planning both at the beginning of the trial and during recruitment. More time can aid in proactively identifying and mitigating risks which can reduce reactive problem-solving and associated heavy workload. The additional time can also provide the flexibility to take bold actions in response to recruitment challenges, ultimately increasing the likelihood of successful recruitment.
If you need to communicate information, consider the most efficient method of delivery (email, message, meeting etc.) and who actually needs to be included.	100.0 %	0.0 %	0.0 %	Include	N/A	N/A	Include without change	No change from original
Focus meetings around objectives with a clear agenda and action points. Tip: Rotate meeting Chairs (e.g., in Trial Managers' meetings) so all managers practice effective meeting management.	71.4 %	0.0 %	28.6 %	Include	N/A	N/A	Include without change	No change from original
Where tasks require urgent attention in the office, establish an agreed staff rota, while respecting flexible arrangements. Staff working in the office can collectively manage urgent administrative tasks on days when other colleagues are working remotely. Thus, promoting equality in hybrid working.	57.1 %	28.6 %	14.3 %	No consensus	Where tasks require urgent attention in the office, establish an agreed staff rota, while respecting flexible arrangements. Staff working in the office can collectively manage urgent administrative tasks on days when other colleagues are working remotely.	60 %	Include without change	No change from first revision
Working overtime should be monitored. Consider allowing greater flexibility in taking Time Off In Lieu (TOIL) of additional hours worked, regardless of staff grade/level. Emphasise to staff that TOIL does not replace annual leave in terms of benefits to wellbeing.	71.4 %	14.3 %	14.3 %	Include	N/A	N/A	Include without change	No change from original
Consider adopting a flexi-time arrangement; a practice often enforced in the public sector to give staff greater autonomy in managing their time – linked to greater job satisfaction. With flexi-time, staff can be allowed to work flexibly and reclaim up to two days of TOIL per month.	71.4 %	14.3 %	14.3 %	Include	N/A	N/A	Update and include	Consider adopting a flexi-time arrangement; a practice often enforced in the public sector to give staff greater autonomy in managing their time – linked to greater job satisfaction. With flexi-time, staff can be allowed to work flexibly and reclaim a pre-agreed amount of overtime per month.
Consider the merit of implementing a 4-day working week. Staff feedback indicates that this change would bring a significant positive impact on most CTU staff's wellbeing. Any such changes require engaging with the relevant institution leads and should only be adopted when in line with organisational policy.	28.6 %	42.9 %	28.6 %	No consensus	Consider the merit of implementing a 4-day working week. Staff feedback indicates that this change would bring a significant positive impact on most CTU staff's wellbeing, boost productivity and commitment. Any such changes require engaging with the relevant institution leads and should only be adopted when in line with organisational policy.	55 %	Update and include	Consider engaging with the appropriate organisation leads to explore the implementation of a four-day working week. Staff feedback indicates that this change would bring a significant positive impact on most CTU staff's wellbeing, boost productivity and commitment. Any such changes require engaging with the relevant institution leads and should only be adopted when in line with organisational policy.
Contributing to impactful research is important to career satisfaction in CTU staff. Periodically share impact reflections/stories on completed trials. Tip: Dissemination can be made via group meetings, key results/an impact statement can also be circulated via email.	85.7 %	14.3 %	0.0 %	Include	N/A	N/A	Include without change	No change from original
Budget development should evidence consideration of appropriate staff time allocation.	85.7 %	14.3 %	0.0 %	Include	N/A	N/A	Update and include	To prevent underbudgeting for staff costs, budget development should evidence consideration of appropriate staff time allocation.
Develop budgets for new grants with input from all relevant teams (e.g., data, IT, trial management) across the main job roles to ensure feasibility of the proposed resources.	85.7 %	14.3 %	0.0 %	Include	N/A	N/A	Include without change	No change from original
Resistance from investigators unfamiliar with CTUs towards cost allocation highlights the importance of making them aware of the role of the CTU and the significance of appropriately funded trials.	57.1 %	28.6 %	14.3 %	No consensus	To ensure investigators understand the importance of accurate costings, make them aware of the role, and advantages of working with a CTU as well as the significance of appropriately funded trials.	53 %	Exclude	N/A
Host knowledge-exchange workshops with study teams from different CTUs and funding bodies to better understand the budget development process and justification of resources.	57.1 %	14.3 %	28.6 %	No consensus	No changes.	67 %	Update and include	Host knowledge-exchange workshops with different CTUs and funding bodies to better understand trial delivery, budget development and justification of resources.
At end of study close-out procedures, reflect on actual staff and non-staff costs (including those sourced from other budgets) to inform future budgeting decisions.	100.0 %	0.0 %	0.0 %	Include	N/A	N/A	Include without change	No change from original
Where funding permits, core staff members (e.g., administrators, communication managers etc.) who are not trial-bound, could be employed to work across trials and provide support easing some workloads.	71.4 %	28.6 %	0.0 %	Include	N/A	N/A	Include without change	No change from original
Stand firm in your position when your experience indicates that a trial is underbudgeted. If collective pushback occurs, it could prevent the subsequent delivery of projects fraught with commonly occurring issues such as failure to meet milestones and staff turnover.	85.7 %	14.3 %	0.0 %	Include	N/A	N/A	Include without change	No change from original
**GROWTH**								
Consider introducing a no-meeting time initiative when training and career development is encouraged. Allow for flexibility in how/when this time is taken (e.g., opt for a monthly minimum quota rather than a fixed day).	100.0 %	0.0 %	0.0 %	Include	N/A	N/A	Update and include	Introduce a no-meeting time initiative when training and career development is encouraged. Allow for flexibility in how/when this time is taken (e.g., opt for a monthly minimum quota rather than a fixed day).
Initiatives such as CTU-wide protected no-meeting time should be respected and honoured across all levels/grades. Tips: Use this time to attend training or complete other career development activities. - Support the inclusion of protected no-meeting time in staff signatures and online diaries to promote wider awareness.	86.0 %	14.0 %	0.0 %	Include	N/A	N/A	Include without change	No change from original
Create and maintain a directory of CTU staff expertise (i.e., names and skill(s) they are experienced in) so colleagues can approach experts directly.	57.0 %	28.5 %	14.5 %	No consensus	Create and maintain a directory of CTU and/or team staff expertise (i.e., names and skill(s) they are experienced in) so colleagues can approach experts directly. Tip: Consider including this in a CTU Induction List and update during staff annual reviews.	77 %	Update and include	Create a directory of CTU and/or team staff expertise (i.e., names and skill(s) they are experienced in) so colleagues can approach experts directly.
Peer-to-peer support (i.e., a buddy system) could help knowledge exchange and career development.	85.7 %	14.3 %	0.0 %	Include	N/A	N/A	Include without change	No change from original
Experienced staff members (on a rota) to host drop-in sessions that colleagues can access to have their queries resolved.	71.4 %	28.6 %	0.0 %	Include	N/A	N/A	Update and include	Experienced staff members (on a rota) to host drop-in sessions that colleagues can access to have their queries resolved. It is important to foster an atmosphere which encourages colleagues to ask questions.
Support and encourage staff across all levels to block time to give a talk on a topic they are experienced in to share their learning.	42.9 %	42.9 %	14.3 %	No consensus	It is important to recognise the skillset of staff across all levels and empower them to share their expertise on specific aspects of clinical trials (e.g., host “lunch n' learn” sessions).	82 %	Include without change	No change from first revision
Create a Microsoft Teams channel/other online teamwork platform community where queries can be posted and answered by colleagues.	71.4 %	14.3 %	14.3 %	Include	N/A	N/A	Include without change	No change from original
Staff should attend training on all aspects of work including (but not limited to), giving feedback, project management, mental health, and wellbeing, as well as technical/task-specific skill training. - Consider attending mental health training; it can increase self-awareness, understanding of others and improve team performance.	57.1 %	28.6 %	14.3 %	No consensus	No changes	100 %	Include without change	No change from original
Create and maintain centralised resource/directory with all training workshops/courses available. Tips: This could be a Teams channel or a SharePoint spreadsheet that staff can post on and find available training opportunities. - Where attendance at training is not feasible for all, designated volunteers (on a rota) can attend and feed back to the wider team on key learnings. - Ask staff to log brief feedback and attendance at training in a centralised resource to aid colleagues in deciding whether a session would be beneficial to attend.	71.4 %	28.6 %	0.0 %	Include	N/A	N/A	Include without change	No change from original
Introduce practical cross-disciplinary training exercises at group meetings. Tips: Staff on a rota could present a study and ask colleagues to identify challenges/solutions. The study could be a new grant, thus using this as an opportunity to problem-solve as a team, or a published clinical trial. 1 a multiple-choice anonymous polling for maximum learning benefits and to reduce evaluation anxiety. - Similarly ask colleagues to solve a problem/achieve a task using the process outlined in a SOP. Consider who should be involved, when and how.	42.9 %	42.9 %	14.3 %	No consensus	Introduce cross-disciplinary training exercises at group meetings. Tips: - Staff on a rota could present a study and ask colleagues to identify challenges/solutions. The study could be a new grant or a published clinical trial. Include a multiple-choice anonymous polling for maximum learning benefits and to reduce evaluation anxiety. - Similarly ask colleagues to solve a problem/achieve a task using the process outlined in a SOP. Consider who should be involved, when and how.	20 %	Exclude	N/A
Host lunchtime seminars to cover a variety of topics, such as examples of successful recruitment strategies, SAE handling etc., to facilitate sharing of expertise in a hybrid working environment.	42.9 %	42.9 %	14.3 %	No consensus	Host mini seminars (e.g. “Tips in ten”) to cover a variety of topics, such as examples of successful trial recruitment strategies, SAE handling etc., to facilitate sharing of expertise in a hybrid working environment.	90 %	Include without change	No change from first revision
Where other strategies have been unsuccessful, consider encouraging new employees who are recent graduates to temporarily spend more time in the office so they are more exposed to established ways of working and code of conduct. Adopt an individualised approach based on people's needs and learning styles.	14.3 %	71.4 %	14.3 %	No consensus	Consider encouraging new employees, with limited trial or work experience, to initially spend more time in the office, allowing them exposure to established ways of working and code of conduct. Adopt an individualised approach based on people's needs and learning styles.	60 %	Exclude	N/A
Establish a CTU-specific mentorship scheme. Participant feedback has indicated that equivalent research/academic programmes do not fully capture the responsibilities and career trajectory of CTU staff and in some cases are not even available to CTUs.	85.7 %	14.3 %	0.0 %	Include	N/A	N/A	Update and include	Establish a CTU-specific mentorship scheme or encourage CTU staff to join an institution-based scheme, where available. Participant feedback has indicated that equivalent research/academic programmes do not fully capture the responsibilities and career trajectory of CTU staff and in some cases are not even available to CTUs.
Establish a CTU-based reverse mentorship programme to support new line managers as well as senior leaders, who may benefit from being exposed to different employee backgrounds and experiences. Ensure mentors-mentees do not belong to the same CTU team.	71.4 %	14.3 %	14.3 %	Include	N/A	N/A	Update and include	Similarly, consider establishing a CTU-based reverse mentorship programme to support new line managers as well as senior leaders, who may benefit from being exposed to different employee backgrounds and experiences. Ensure mentors-mentees do not belong to the same trial team, or, at the very least, are not bound by a line management relationship.
Encourage line managers across all levels/grades to attend line managers'/leadership training.	85.7 %	14.3 %	0.0 %	Include	N/A	N/A	Include without change	No change from original
Where line management training is offered by the University but not available to CTU staff, consider engaging with the relevant University leads to request access is granted. Emphasise how integral it is for career development at CTUs.	42.9 %	28.6 %	28.6 %	No consensus	Where line management training is offered by the host organisation (e.g. University or NHS) but not available to CTU staff, engage with the relevant organisational leads to request access.	100 %	Include without change	No change from first revision
Where the CTU leads the development of line management training consider the following: Participant feedback indicates that staff connect with and retain information that included stories/experiences that they can relate to. – include content such as active listening skills, managing different personalities etc. - Break training into separate modules <20 min; so, staff can refer back as and when needed without having to sit through a long module. - Training should be recorded and online options offered for ease of access.	71.4 %	14.3 %	14.3 %	Include	N/A	N/A	Update and include	Where the CTU leads the development of line management training consider the following: - Participant feedback indicates that staff connect with and retain information better when that includes stories/experiences they can relate to. – Include content such as active listening skills, managing different personalities etc. - Break training into separate modules, <20 min long, so, staff can refer back as and when needed without having to sit through a long module. - Training should be recorded and online options offered for ease of access.
Where staff are required to complete timesheets, they could be allowed to put down any time spent helping colleagues as trial work. If all staff follow this practice, helping time would be evenly spread across trials.	14.3 %	42.9 %	42.9 %	No consensus	Where staff are required to complete timesheets, they could be encouraged to put down any time spent helping colleagues as trial work. If all staff follow this practice, time to help colleagues would be evenly spread across trials and would prevent unfair use of resources.	55 %	Exclude	N/A

The consensus workshop was attended by 15 consensus panel members (n = 9 participants, n = 6 SAG members), plus two researchers (ER, SSH). The consensus panel reviewed all 67 recommendations. The 38 items with consensus to include were reviewed for comprehension, 12 were agreed to ‘include without change’, with remaining items under-going minor edits. The 29 items with no consensus were formally rated to ‘include’ (n = 11), ‘exclude’ (n = 6), or ‘update and include’ (n = 12) following discussions. Therefore, 61 items reached agreement to include as core statements in the final guidance.

### 3.4 Stage 3: Guidance finalisation

The sixty-one wellbeing recommendations that reached agreement for inclusion in the final document related to four aspects of flourishing, communication (n = 21), the environment (n = 17), opportunities for growth (n = 15), and acknowledgement (n = 8). During the iterative review process the need for additional detail to be included in the guidance was emphasised to provide context and aid implementation, including key take-home messages, representative participant quotes, and additional useful tips.

The consensus panel discussed that whilst some statements may be perceived as common sense by organisations who have already adopted these practices, for others they may be novel and should not be overlooked. As such, the guidance is developed as a foundation tool for CTUs to adapt to their own needs. Panel members emphasised the need for sharing of good practice, in terms of wellbeing initiatives, among CTUs and identified a lack of resources and time needed to facilitate sharing of expertise as barriers to implementation.

## 4 Discussion

With the aim of developing guidance to support CTU staff to flourish in the workplace, focus groups were conducted to identify recommendations to support the wellbeing challenges experienced by CTU staff. Through a consensus panel survey and workshop, agreement was reached on which recommendations should be included in the Flourishing As Clinical Trial Staff (FACTS) guidance.

The focus group discussions built upon the results of a recent national survey of 484 UKCRC CTU staff [36]. Participants discussed job demands and suggested possible resources specific to their experience of working in a CTU. The suggested demands/resources were relevant to four aspects of work at CTUs, the Environment, Communication, Growth, and Acknowledgement. These factors highlight the importance of the concept of flourishing when considering how best to support the wellbeing of CTU staff [23,34,40,41] and emphasise the need for wellbeing guidance which specifically addresses these demands.

With regards to the environment, participants discussed issues relating to time and meeting management, autonomy including flexible working, and work-life balance. Indeed, recent research on academics working in a University setting has shown that flexible working can support work-life balance and be viewed by employees as a form of organisational support which facilitates flourishing [42]. Participants identified the importance of supporting CTU staff to set aside time for diary/priority planning, actively monitoring overtime, and supporting flexible and autonomous working. Many of the FACTS recommendations can be readily implemented in CTUs, whereas others will be policy specific, for example TOIL. Nonetheless, these should not be overlooked given the potential promise of these strategies for promoting workplace wellbeing and productivity [[43], [44], [45]].

Demands relating to communicating within a broad inter-disciplinary team were associated with working in a CTU, with specific issues relating to inter-team working, role expectations and transparency in decision making. Previous research with staff working in healthcare settings [41] and manufacturing organisations [46,47] has identified the importance of actively supporting positive relationships through transparent decision making via regular meetings and by developing trusting relationships. Here, CTU staff emphasised the need for all staff to feel listened to and involved, where possible, in decision making. Strategies to raise awareness of all the job roles (e.g., statisticians, Quality Assurance, data management) within a CTU could bring several benefits including increasing respect and setting realistic expectations. This in turn may improve time/project management demands, and increase unit-wide recognition of individuals effort and contributions.

Growth and development has been identified as key to flourishing for those working in healthcare settings [48], and here we have highlighted its importance for CTU staff. Participants felt that simple strategies could be implemented, such as promoting sharing of multi-disciplinary expertise, which is inherent with CTU working, and offering mentorship/buddying schemes. However, a re-occurring issue which should be considered is providing protected time for training and taking a wider view on what skills should be developed in CTU staff, such as supporting soft skill development (e.g., time management, interpersonal skills, mental health literacy) as well as clinical research and technical skills.

Finally, receiving appropriate acknowledgement was something considered by participants as a factor of working in a CTU which should be addressed. This is supported by research with healthcare professionals which has identified acknowledgement as an important aspect of workplace flourishing [49]. Acknowledgement was thought to be important regardless of the scale of gesture. Furthermore, it was highlighted that CTUs should prioritise training in delivering appropriate feedback to colleagues. Research shows this is crucial for enabling individuals to assess their progress, address performance uncertainties, and refine their development goals [50].

Identifying solutions to the demands associated with working in a CTU enables us to develop unique and targeted guidance which provides a clear solution-focussed approach to supporting CTU staff wellbeing which is not currently available. Although existing workplace wellbeing guidelines, such as the Farmer-Stevenson review [38], emphasise the importance of meeting Mental Health Core Standards, there is a lack of detail as to how to achieve this. Indeed, as identified in the Farmer-Stevenson review, practical solutions should be developed through collaboration with employees, since mutually agreed solutions offer the best chance of buy-in and sustainability. Similarly, NICE mental health at work guidelines highlight that wellbeing guidance should be relevant to individual organisations, with a focus on preventative solutions that reduce workplace stressors and help employees cope with the demands placed on them [39].

The recommendations developed in the FACTS guidance are designed to promote a change in the way in which CTUs consider staff wellbeing and recognise the demands placed upon staff. It is hoped that the implementation of this guidance will support with addressing the retention challenges facing CTUs and therefore potentially support efficient trial delivery. Future research is required to establish such relationships.

The FACTS guidance has been created with all CTU job roles in mind. The guidance recommendations include changes that can be accomplished at an individual, team/managerial or unit level. To ensure effectiveness, it is important to engage staff at all levels in wellbeing decisions, with senior staff taking a ‘lead by example’ approach and supporting engagement with relevant wellbeing training where appropriate. It is acknowledged that not all recommendations will be relevant to, or be able to be adapted by, all CTUs. Nonetheless as identified through this research, it is important that we begin to challenge standard hesitant responses to more radical changes and encourage discussions with operational/institutional managers, Government policymakers, and major funding bodies who often underlie the contracts of CTU workers through external grant money. At a more localised level, it is recognised that most UKCRC-registered CTUs operate within a wider organisation, such as a University or Trust. In some cases, CTUs are limited by the constraints set by their wider organisation when implementing change, and it is important that effective liaisons are supported between the two to bring sustainable change. We also recognise that several CTUs already adopt excellent initiatives to support staff wellbeing, and these are unlikely to be comprehensively covered here. As such, it is suggested that the guidance is regularly reviewed, updated, and used as a platform for future cross unit discussions and sharing of good practice. Establishing local CTU wellbeing groups and tailoring organisational wide (e.g., university or healthcare trust) mental health resources to CTU staff needs is recommended.

The implications of this work should be considered in light of its limitations, particularly the fact that individuals in Trial Management roles were most represented in the focus groups. Consequently, the recommendations developed may be especially relevant to those in similar roles. However, the wellbeing challenges discussed were generally broad and not specific to any one role, which likely minimises any negative impact on generalisability. Additionally, Trial Management teams typically constitute the largest group within CTUs. That said, individual preferences vary, and the wellbeing recommendations proposed may resonate more with some individuals than others. Notably, the primarily qualitative approach used here may not fully capture the perspectives of the broader population. However, because the wellbeing challenges identified in focus groups align closely with findings from a national survey of 484 CTU staff [36], any potential limitations due to the qualitative nature of the study are likely minimal. Future research is recommended to provide a comprehensive examination of the factors that contribute to successful implementation, including considering the resources required, risks, benefits, and mechanisms of action, at individual and unit wide level.

## 5 Conclusion

Our findings support a flourishing framework in relation to CTU staff wellbeing which is underpinned by the following pillars: positive interpersonal relationships at work, autonomy over one's environment including flexible working patterns and location, manageable workloads, opportunities for personal and professional development, effort and outcome recognised and appreciated, and measures taken proactively to boost employees' mental and physical wellbeing. By implementing the recommendations reported here, CTUs may not only increase staff flourishing but could also increase retention, enhance trial efficiency and data quality due to increased motivation and reduced staff turnover. Recognising limited time and resources for considering wellbeing in CTUs, the FACTS guidance provides adaptable recommendations to address local needs. Although the guidance was created specifically for staff working in UKCRC-registered CTUs, the findings are likely to be applicable to those working in clinical research organisations more broadly. The guidance document, incorporating infographic and textual descriptions, will be made available for download on the Nottingham Clinical Trials Unit FACTS webpage: https://www.nctu.ac.uk/Our-Research/Methodology/Current-Studies/FACTS.aspx.

## CRediT authorship contribution statement

**Sophie S. Hall:** Writing – original draft, Formal analysis, Data curation, Supervision, Funding acquisition, Conceptualization, Methodology. **Evgenia Riga:** Data curation, Project administration, Methodology, Writing – review & editing, Formal analysis. **Eleanor J. Mitchell:** Conceptualization, Supervision, Writing – review & editing, Funding acquisition. **Louise Thomson:** Funding acquisition, Conceptualization, Supervision, Writing – review & editing. **Jodi Taylor:** Writing – review & editing, Conceptualization, Supervision, Funding acquisition. **Lucy Carr:** Funding acquisition, Writing – review & editing, Supervision, Conceptualization. **Pamela Hagan:** Funding acquisition, Writing – review & editing, Supervision, Conceptualization. **Kirsty Sprange:** Supervision, Conceptualization, Funding acquisition, Writing – review & editing.

## Funding sources

This study was funded by the 10.13039/501100000272NIHR, [Efficient/innovative delivery of 10.13039/501100000272NIHR research 2022 (NIHR152373)]. The views expressed are those of the authors and not necessarily those of the NIHR or the Department of Health and Social Care.

## Declaration of competing interest

The authors declare the following financial interests/personal relationships which may be considered as potential competing interests:Sophie Hall reports financial support was provided by 10.13039/501100000272National Institute for Health and Care Research. If there are other authors, they declare that they have no known competing financial interests or personal relationships that could have appeared to influence the work reported in this paper.

## Data Availability

Data are available from the authors upon reasonable request and with the permission of the study team.

## Glossary

***Flourishing***: Flourishing is a state of thriving, growth, and well-being, where individuals experience fulfilment, purpose, and positive development. It represents the positive aspect of wellbeing
***Environment***: External conditions and social contexts that support or hinder an individual's ability to flourish
***Interpersonal communication***: Meaningful and authentic exchanges between individuals that foster personal growth, deep connections, and a sense of purpose. It involves open, supportive, and empathetic interactions
***Growth***: Continuous development of one's potential, character, and abilities
***Acknowledgement***: Recognition and validation of one's intrinsic worth, efforts, and personal growth by oneself or others
